# 4-(2-Fluoro­phen­yl)-2-meth­oxy-5,6,7,8,9,10-hexa­hydro­cyclo­octa­[*b*]pyridine-3-carbo­nitrile

**DOI:** 10.1107/S1600536814016365

**Published:** 2014-07-23

**Authors:** R. Vishnupriya, J. Suresh, S. Maharani, R. Ranjith Kumar, P. L. Nilantha Lakshman

**Affiliations:** a Department of Physics, The Madura College, Madurai 625 011, India; b Department of Organic Chemistry, School of Chemistry, Madurai Kamaraj University, Madurai 625 021, India; c Department of Food Science and Technology, University of Ruhuna, Mapalana, Kamburupitiya 81100, Sri Lanka

**Keywords:** crystal structure, cyclo­octa­[*b*]pyridine, carbo­nitrile compounds

## Abstract

In the title compound, C_19_H_19_FN_2_O, the cyclo­octene ring adopts a twisted boat–chair conformation. The dihedral angle between the plane of the fluorophenyl substituent and that of the pyridine ring is 76.39 (8)°. The F and *ortho*-H atoms of the fluoro­benzene ring are disordered, with occupancy factors of 0.226 (5) and 0.774 (5). In the crystal, no significant inter­actions are observed between the mol­ecules beyond van der Waals contacts.

## Related literature   

For the biological activities of substituted pyridine derivatives, see: Bossert & Vater (1989 ▶); Bossert *et al.* (1981 ▶); Wang *et al.* (1989 ▶); Alajarin *et al.* (1995 ▶). For similar structures, see: Ramesh *et al.* (2009*a* ▶,*b* ▶).
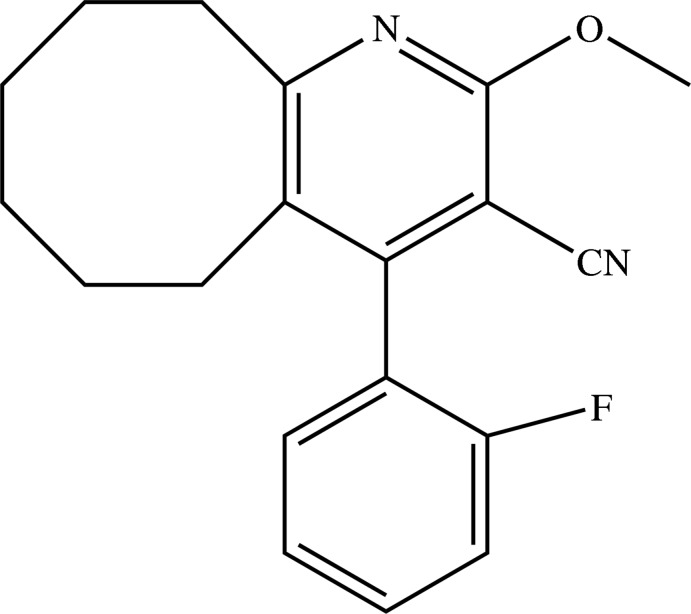


## Experimental   

### 

#### Crystal data  

C_19_H_19_FN_2_O
*M* *_r_* = 310.36
Monoclinic, 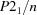

*a* = 9.5219 (3) Å
*b* = 13.8808 (4) Å
*c* = 12.1140 (3) Å
β = 97.829 (1)°
*V* = 1586.20 (8) Å^3^
*Z* = 4
Mo *K*α radiation
μ = 0.09 mm^−1^
*T* = 293 K
0.28 × 0.25 × 0.23 mm

#### Data collection  

Bruker Kappa APEXII diffractometer
Absorption correction: multi-scan (*SADABS*; Sheldrick, 1996 ▶) *T*_min_ = 0.977, *T*_max_ = 0.981
37690 measured reflections
3475 independent reflections
2812 reflections with *I* > 2σ(*I*)
*R*_int_ = 0.027

#### Refinement  

*R*[*F* ^2^ > 2σ(*F* ^2^)] = 0.053
*wR*(*F* ^2^) = 0.155
*S* = 1.08
3475 reflections
220 parameters
10 restraints
H-atom parameters constrained
Δρ_max_ = 0.65 e Å^−3^
Δρ_min_ = −0.61 e Å^−3^

### 

Data collection: *APEX2* (Bruker, 2004 ▶); cell refinement: *SAINT* (Bruker, 2004 ▶); data reduction: *SAINT*; program(s) used to solve structure: *SHELXS97* (Sheldrick, 2008 ▶); program(s) used to refine structure: *SHELXL97* (Sheldrick, 2008 ▶); molecular graphics: *PLATON* (Spek, 2009 ▶); software used to prepare material for publication: *SHELXL97*.

## Supplementary Material

Crystal structure: contains datablock(s) global, I. DOI: 10.1107/S1600536814016365/zq2223sup1.cif

Structure factors: contains datablock(s) I. DOI: 10.1107/S1600536814016365/zq2223Isup2.hkl

Supporting information file. DOI: 10.1107/S1600536814016365/zq2223Isup3.cml

CCDC reference: 1013943

Additional supporting information:  crystallographic information; 3D view; checkCIF report

## Figures and Tables

**Fig. 1.**
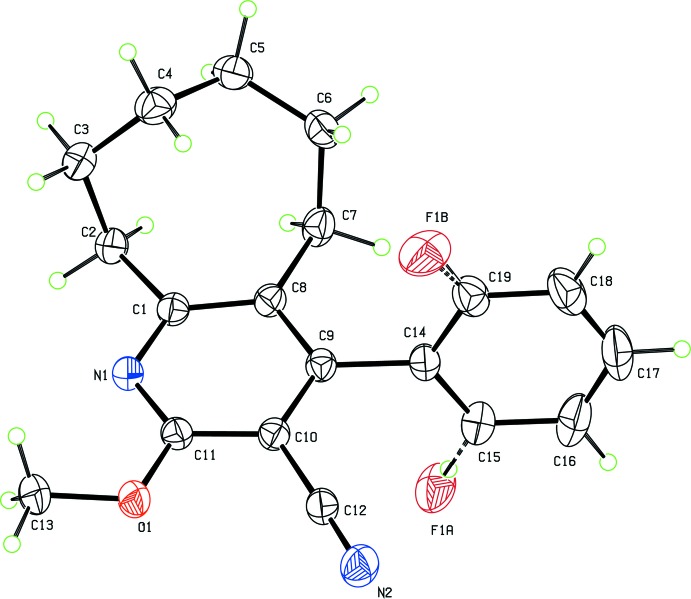
The molecular structure of the title compound showing 30% probability displacement ellipsoids and the atom-numbering scheme.

## supplementary crystallographic information

### S1. Comment

The synthesis of hydrogenated compounds has been extensively studied due to their interesting biological properties. For example, derivatives of 1,4-dihydropyridine exhibit high biological activities as calcium channel blockers (Bossert *et al.*, 1981) and as calcium agonists or antagonists (Bossert & Vater, 1989; Wang *et al.*,1989; Alajarin *et al.*, 1995). Our interest in preparing pharmacologically active pyridine-related compounds led us to the title compound, derived from a 1,4-dihydropyridine and we have undertaken X-ray crystal structure determination of substituted pyridine scaffolds in order to establish its molecular conformation.

The molecular structure of the title compound is shown in Fig 1. The cyclooctane ring (C1–C8) adopts twisted boat chair conformation. The central pyridine component is planar, with a maximum deviation from the mean plane that of 0.0207 (1) Å for atom C1. The phenyl substituent at C9 of the pyridine ring has a (+) synclinal conformation, which is evidenced by the C15–C14–C9–C10 torsion angle 77.10 (18)°. The shortening of the C–N distances [1.347 (2) and 1.312 (2) Å] and the opening of the N1–C11–C10 angle [122.98 (16)°] may be attributed to the size of the substituent at C1. There is a long C*sp*^2^—C*sp*^1^ bond (C10–C12 ═1.433 (3) Å), due to conjugation as found in similar related structures (Ramesh *et al.*, 2009*a*, 2009*b*). The dihedral angle between the pseudo-axial phenyl substituent and the plane of the pyridine ring is 76.39 (8)°. Due to conjugation, the bond length C11—O1 (1.342 (2) Å) is shorter than the bond length C13—O1 (1.434 (2) Å).

No significant intermolecular hydrogen bonds, π—π stacking interactions between neighboring aromatic rings or C—H···π interactions towards them are observed.

### S2. Experimental

A mixture of cyclooctanone (1 mmol), 2-fluorobenzaldehyde (1 mmol) and malononitrile (1 mmol) were taken in methanol (10 ml) to which lithium ethoxide (1 equiv) was added. The reaction mixture was heated under reflux for 2–3 h. After completion of the reaction (TLC), the reaction mixture was poured into crushed ice and extracted with ethyl acetate. The excess solvent was removed under vacuum and the residue was subjected to column chromatography using petroleum ether/ethyl acetate mixture (95:5 *v*/*v*) as eluent to obtain pure product. Melting point: 161–162 °C, yield: 67%.

### S3. Refinement

Crystal data, data collection and structure refinement details are summarized in Table 1. H atoms were placed in calculated positions and allowed to ride on their carrier atoms with C—H = 0.93 (aromatic CH), 0.96 (methyl CH_3_) and 0.97 Å (methylene CH_2_). Isotropic displacement parameters for H atoms were calculated as *U*_iso_ = 1.5*U*_eq_(C) for CH_3_ groups and *U*_iso_ = 1.2*U*_eq_(carrier atom) for all other H atoms. The F and H atoms of the fluorobenzene rings are disordered over two sets of sites in the ratio 0.226(): 0.774 (5).

### Crystal data

**Table:**

C_19_H_19_FN_2_O	*F*(000) = 656
*M**_r_* = 310.36	*D*_x_ = 1.300 Mg m^−^^3^
Monoclinic, *P*2_1_/*n*	Mo *K*α radiation, λ = 0.71073 Å
Hall symbol: -P 2yn	Cell parameters from 2000 reflections
*a* = 9.5219 (3) Å	θ = 2–27°
*b* = 13.8808 (4) Å	µ = 0.09 mm^−^^1^
*c* = 12.1140 (3) Å	*T* = 293 K
β = 97.829 (1)°	Block, colourless
*V* = 1586.20 (8) Å^3^	0.28 × 0.25 × 0.23 mm
*Z* = 4	

### Data collection

**Table:**

Bruker Kappa APEXII diffractometer	3475 independent reflections
Radiation source: fine-focus sealed tube	2812 reflections with *I* > 2σ(*I*)
Graphite monochromator	*R*_int_ = 0.027
Detector resolution: 0 pixels mm^-1^	θ_max_ = 27.0°, θ_min_ = 2.2°
ω and φ scans	*h* = −12→12
Absorption correction: multi-scan (*SADABS*; Sheldrick, 1996)	*k* = −17→17
*T*_min_ = 0.977, *T*_max_ = 0.981	*l* = −15→15
37690 measured reflections	

### Refinement

**Table:**

Refinement on *F*^2^	Secondary atom site location: difference Fourier map
Least-squares matrix: full	Hydrogen site location: inferred from neighbouring sites
*R*[*F*^2^ > 2σ(*F*^2^)] = 0.053	H-atom parameters constrained
*wR*(*F*^2^) = 0.155	*w* = 1/[σ^2^(*F*_o_^2^) + (0.0648*P*)^2^ + 0.7644*P*] where *P* = (*F*_o_^2^ + 2*F*_c_^2^)/3
*S* = 1.08	(Δ/σ)_max_ < 0.001
3475 reflections	Δρ_max_ = 0.65 e Å^−^^3^
220 parameters	Δρ_min_ = −0.61 e Å^−^^3^
10 restraints	Extinction correction: *SHELXL97* (Sheldrick, 2008), Fc^*^=kFc[1+0.001xFc^2^λ^3^/sin(2θ)]^-1/4^
Primary atom site location: structure-invariant direct methods	Extinction coefficient: 0.010 (2)

### Special details

**Table:**

Geometry. All e.s.d.'s (except the e.s.d. in the dihedral angle between two l.s. planes) are estimated using the full covariance matrix. The cell e.s.d.'s are taken into account individually in the estimation of e.s.d.'s in distances, angles and torsion angles; correlations between e.s.d.'s in cell parameters are only used when they are defined by crystal symmetry. An approximate (isotropic) treatment of cell e.s.d.'s is used for estimating e.s.d.'s involving l.s. planes.
Refinement. Refinement of *F*^2^ against ALL reflections. The weighted *R*-factor *wR* and goodness of fit *S* are based on *F*^2^, conventional *R*-factors *R* are based on *F*, with *F* set to zero for negative *F*^2^. The threshold expression of *F*^2^ > σ(*F*^2^) is used only for calculating *R*-factors(gt) *etc*. and is not relevant to the choice of reflections for refinement. *R*-factors based on *F*^2^ are statistically about twice as large as those based on *F*, and *R*- factors based on ALL data will be even larger.

### Fractional atomic coordinates and isotropic or equivalent isotropic displacement parameters (Å^2^)

**Table:**

	*x*	*y*	*z*	*U*_iso_*/*U*_eq_	Occ. (<1)
C1	0.48826 (19)	0.14942 (13)	0.47836 (14)	0.0386 (4)	
C2	0.5667 (2)	0.18410 (14)	0.38693 (15)	0.0454 (4)	
H2A	0.5635	0.1344	0.3303	0.054*	
H2B	0.6653	0.1942	0.4170	0.054*	
C3	0.5073 (2)	0.27694 (16)	0.33279 (17)	0.0537 (5)	
H3A	0.5695	0.2983	0.2806	0.064*	
H3B	0.4156	0.2631	0.2903	0.064*	
C4	0.4896 (2)	0.35887 (16)	0.41229 (19)	0.0575 (5)	
H4A	0.4163	0.3413	0.4569	0.069*	
H4B	0.4566	0.4152	0.3689	0.069*	
C5	0.6229 (3)	0.38643 (15)	0.49078 (18)	0.0566 (5)	
H5A	0.7047	0.3636	0.4588	0.068*	
H5B	0.6286	0.4562	0.4943	0.068*	
C6	0.6324 (3)	0.34781 (16)	0.60938 (18)	0.0593 (6)	
H6A	0.5458	0.3651	0.6384	0.071*	
H6B	0.7098	0.3807	0.6546	0.071*	
C7	0.6549 (2)	0.23933 (16)	0.62511 (16)	0.0496 (5)	
H7A	0.7308	0.2200	0.5839	0.059*	
H7B	0.6871	0.2274	0.7034	0.059*	
C8	0.52873 (18)	0.17526 (13)	0.58972 (14)	0.0384 (4)	
C9	0.45081 (18)	0.13641 (12)	0.66895 (14)	0.0370 (4)	
C10	0.33770 (18)	0.07500 (12)	0.63392 (14)	0.0380 (4)	
C11	0.30274 (19)	0.05759 (13)	0.51926 (14)	0.0396 (4)	
C12	0.2597 (2)	0.02745 (14)	0.71119 (15)	0.0438 (4)	
C13	0.1466 (3)	−0.01094 (17)	0.37142 (17)	0.0565 (5)	
H13A	0.0670	−0.0539	0.3596	0.085*	
H13B	0.2237	−0.0373	0.3376	0.085*	
H13C	0.1203	0.0505	0.3385	0.085*	
C14	0.48547 (16)	0.15856 (14)	0.79050 (14)	0.0421 (4)	
C15	0.55536 (16)	0.09561 (12)	0.86460 (16)	0.0557 (5)	
H15	0.5783	0.0356	0.8380	0.067*	0.226 (5)
F1B	0.3841 (3)	0.3087 (4)	0.7588 (5)	0.115 (4)	0.226 (5)
C16	0.5944 (2)	0.1143 (2)	0.97544 (19)	0.0741 (8)	
H16	0.6419	0.0686	1.0227	0.089*	
C17	0.5606 (3)	0.2033 (3)	1.0142 (2)	0.0807 (9)	
H17	0.5861	0.2185	1.0891	0.097*	
C18	0.4901 (3)	0.2696 (2)	0.9439 (2)	0.0737 (8)	
H18	0.4672	0.3296	0.9708	0.088*	
C19	0.4532 (2)	0.24716 (17)	0.83331 (18)	0.0571 (5)	
H19	0.4054	0.2926	0.7859	0.069*	0.774 (5)
F1A	0.5857 (2)	0.00848 (12)	0.82521 (14)	0.0769 (8)	0.774 (5)
N1	0.37525 (17)	0.09332 (11)	0.44417 (12)	0.0417 (4)	
N2	0.1995 (2)	−0.01308 (16)	0.77174 (16)	0.0628 (5)	
O1	0.18988 (15)	0.00092 (11)	0.48868 (11)	0.0518 (4)	

### Atomic displacement parameters (Å^2^)

**Table:**

	*U*^11^	*U*^22^	*U*^33^	*U*^12^	*U*^13^	*U*^23^
C1	0.0420 (9)	0.0375 (9)	0.0364 (9)	0.0037 (7)	0.0055 (7)	0.0002 (7)
C2	0.0526 (11)	0.0467 (10)	0.0383 (9)	−0.0006 (8)	0.0116 (8)	−0.0020 (8)
C3	0.0626 (12)	0.0571 (12)	0.0401 (10)	−0.0043 (10)	0.0021 (9)	0.0066 (9)
C4	0.0647 (13)	0.0491 (11)	0.0588 (13)	0.0085 (10)	0.0090 (10)	0.0078 (10)
C5	0.0719 (14)	0.0432 (10)	0.0570 (12)	−0.0088 (10)	0.0170 (10)	−0.0035 (9)
C6	0.0699 (14)	0.0607 (13)	0.0486 (11)	−0.0243 (11)	0.0127 (10)	−0.0139 (10)
C7	0.0431 (10)	0.0664 (13)	0.0378 (9)	−0.0106 (9)	0.0002 (7)	0.0045 (9)
C8	0.0380 (9)	0.0400 (9)	0.0366 (8)	0.0014 (7)	0.0025 (7)	0.0022 (7)
C9	0.0380 (9)	0.0381 (8)	0.0340 (8)	0.0039 (7)	0.0019 (6)	0.0003 (7)
C10	0.0405 (9)	0.0383 (9)	0.0349 (8)	0.0019 (7)	0.0043 (7)	0.0005 (7)
C11	0.0427 (9)	0.0374 (9)	0.0374 (9)	−0.0001 (7)	0.0010 (7)	−0.0009 (7)
C12	0.0447 (10)	0.0489 (10)	0.0377 (9)	−0.0029 (8)	0.0058 (7)	−0.0039 (8)
C13	0.0626 (13)	0.0613 (13)	0.0414 (10)	−0.0116 (10)	−0.0081 (9)	−0.0042 (9)
C14	0.0396 (9)	0.0516 (10)	0.0350 (9)	−0.0050 (8)	0.0046 (7)	−0.0034 (7)
C15	0.0576 (12)	0.0679 (14)	0.0404 (10)	0.0027 (10)	0.0024 (9)	0.0012 (9)
F1B	0.133 (8)	0.091 (6)	0.122 (7)	0.054 (5)	0.026 (6)	−0.025 (5)
C16	0.0668 (15)	0.113 (2)	0.0402 (11)	−0.0049 (15)	−0.0012 (10)	0.0109 (13)
C17	0.0796 (17)	0.123 (3)	0.0404 (12)	−0.0294 (17)	0.0112 (11)	−0.0250 (15)
C18	0.0838 (17)	0.0808 (17)	0.0614 (15)	−0.0219 (14)	0.0281 (13)	−0.0317 (14)
C19	0.0610 (13)	0.0617 (13)	0.0516 (11)	−0.0084 (10)	0.0183 (10)	−0.0141 (10)
F1A	0.1046 (16)	0.0693 (12)	0.0548 (11)	0.0386 (10)	0.0034 (9)	0.0068 (8)
N1	0.0484 (9)	0.0409 (8)	0.0350 (7)	−0.0002 (6)	0.0034 (6)	−0.0010 (6)
N2	0.0648 (12)	0.0756 (13)	0.0504 (10)	−0.0147 (10)	0.0164 (9)	0.0009 (9)
O1	0.0554 (8)	0.0587 (8)	0.0388 (7)	−0.0169 (6)	−0.0025 (6)	−0.0006 (6)

### Geometric parameters (Å, º)

**Table:**

C1—N1	1.347 (2)	C9—C14	1.496 (2)
C1—C8	1.398 (2)	C10—C11	1.404 (2)
C1—C2	1.497 (2)	C10—C12	1.433 (3)
C2—C3	1.520 (3)	C11—N1	1.312 (2)
C2—H2A	0.9700	C11—O1	1.342 (2)
C2—H2B	0.9700	C12—N2	1.139 (3)
C3—C4	1.514 (3)	C13—O1	1.434 (2)
C3—H3A	0.9700	C13—H13A	0.9600
C3—H3B	0.9700	C13—H13B	0.9600
C4—C5	1.527 (3)	C13—H13C	0.9600
C4—H4A	0.9700	C14—C15	1.360 (3)
C4—H4B	0.9700	C14—C19	1.385 (3)
C5—C6	1.524 (3)	C15—F1A	1.3459 (10)
C5—H5A	0.9700	C15—C16	1.368 (3)
C5—H5B	0.9700	C15—H15	0.9300
C6—C7	1.529 (3)	F1B—C19	1.3477 (10)
C6—H6A	0.9700	C16—C17	1.375 (4)
C6—H6B	0.9700	C16—H16	0.9300
C7—C8	1.509 (3)	C17—C18	1.367 (4)
C7—H7A	0.9700	C17—H17	0.9300
C7—H7B	0.9700	C18—C19	1.374 (3)
C8—C9	1.399 (2)	C18—H18	0.9300
C9—C10	1.394 (2)	C19—H19	0.9300
			
N1—C1—C8	123.26 (16)	C9—C8—C7	120.56 (15)
N1—C1—C2	114.61 (15)	C10—C9—C8	119.14 (15)
C8—C1—C2	122.11 (16)	C10—C9—C14	118.89 (15)
C1—C2—C3	113.46 (16)	C8—C9—C14	121.97 (15)
C1—C2—H2A	108.9	C9—C10—C11	118.40 (16)
C3—C2—H2A	108.9	C9—C10—C12	122.05 (16)
C1—C2—H2B	108.9	C11—C10—C12	119.52 (16)
C3—C2—H2B	108.9	N1—C11—O1	120.49 (16)
H2A—C2—H2B	107.7	N1—C11—C10	122.98 (16)
C4—C3—C2	115.43 (16)	O1—C11—C10	116.53 (16)
C4—C3—H3A	108.4	N2—C12—C10	177.8 (2)
C2—C3—H3A	108.4	O1—C13—H13A	109.5
C4—C3—H3B	108.4	O1—C13—H13B	109.5
C2—C3—H3B	108.4	H13A—C13—H13B	109.5
H3A—C3—H3B	107.5	O1—C13—H13C	109.5
C3—C4—C5	115.42 (19)	H13A—C13—H13C	109.5
C3—C4—H4A	108.4	H13B—C13—H13C	109.5
C5—C4—H4A	108.4	C15—C14—C19	115.90 (18)
C3—C4—H4B	108.4	C15—C14—C9	122.68 (17)
C5—C4—H4B	108.4	C19—C14—C9	121.36 (18)
H4A—C4—H4B	107.5	F1A—C15—C14	116.92 (17)
C6—C5—C4	115.89 (18)	F1A—C15—C16	118.4 (2)
C6—C5—H5A	108.3	C14—C15—C16	124.7 (2)
C4—C5—H5A	108.3	C14—C15—H15	117.7
C6—C5—H5B	108.3	C16—C15—H15	117.7
C4—C5—H5B	108.3	C15—C16—C17	117.5 (3)
H5A—C5—H5B	107.4	C15—C16—H16	121.3
C5—C6—C7	116.94 (17)	C17—C16—H16	121.3
C5—C6—H6A	108.1	C18—C17—C16	120.7 (2)
C7—C6—H6A	108.1	C18—C17—H17	119.7
C5—C6—H6B	108.1	C16—C17—H17	119.7
C7—C6—H6B	108.1	C17—C18—C19	119.5 (3)
H6A—C6—H6B	107.3	C17—C18—H18	120.2
C8—C7—C6	116.88 (17)	C19—C18—H18	120.2
C8—C7—H7A	108.1	F1B—C19—C18	123.1 (4)
C6—C7—H7A	108.1	F1B—C19—C14	115.2 (4)
C8—C7—H7B	108.1	C18—C19—C14	121.8 (2)
C6—C7—H7B	108.1	C18—C19—H19	119.1
H7A—C7—H7B	107.3	C14—C19—H19	119.1
C1—C8—C9	117.45 (16)	C11—N1—C1	118.64 (15)
C1—C8—C7	121.95 (16)	C11—O1—C13	116.85 (15)
			
N1—C1—C2—C3	87.0 (2)	C10—C9—C14—C15	77.10 (18)
C8—C1—C2—C3	−91.8 (2)	C8—C9—C14—C15	−102.71 (18)
C1—C2—C3—C4	52.0 (2)	C10—C9—C14—C19	−105.84 (18)
C2—C3—C4—C5	54.9 (3)	C8—C9—C14—C19	74.3 (2)
C3—C4—C5—C6	−100.7 (2)	C19—C14—C15—F1A	178.92 (13)
C4—C5—C6—C7	69.8 (3)	C9—C14—C15—F1A	−3.88 (17)
C5—C6—C7—C8	−74.8 (3)	C19—C14—C15—C16	−0.02 (14)
N1—C1—C8—C9	3.2 (3)	C9—C14—C15—C16	177.18 (17)
C2—C1—C8—C9	−178.04 (16)	F1A—C15—C16—C17	−179.13 (17)
N1—C1—C8—C7	−179.21 (17)	C14—C15—C16—C17	−0.2 (2)
C2—C1—C8—C7	−0.4 (3)	C15—C16—C17—C18	0.4 (3)
C6—C7—C8—C1	80.7 (2)	C16—C17—C18—C19	−0.3 (3)
C6—C7—C8—C9	−101.8 (2)	C17—C18—C19—F1B	−179.75 (17)
C1—C8—C9—C10	−0.3 (2)	C17—C18—C19—C14	0.1 (3)
C7—C8—C9—C10	−177.92 (17)	C15—C14—C19—F1B	179.93 (8)
C1—C8—C9—C14	179.53 (16)	C9—C14—C19—F1B	2.7 (2)
C7—C8—C9—C14	1.9 (3)	C15—C14—C19—C18	0.08 (18)
C8—C9—C10—C11	−2.5 (3)	C9—C14—C19—C18	−177.16 (17)
C14—C9—C10—C11	177.63 (16)	O1—C11—N1—C1	−179.64 (16)
C8—C9—C10—C12	175.57 (17)	C10—C11—N1—C1	−0.1 (3)
C14—C9—C10—C12	−4.2 (3)	C8—C1—N1—C11	−3.0 (3)
C9—C10—C11—N1	2.9 (3)	C2—C1—N1—C11	178.14 (16)
C12—C10—C11—N1	−175.29 (17)	N1—C11—O1—C13	−5.3 (3)
C9—C10—C11—O1	−177.58 (16)	C10—C11—O1—C13	175.14 (17)
C12—C10—C11—O1	4.2 (3)		
